# Sperm whale dive behavior characteristics derived from intermediate‐duration archival tag data

**DOI:** 10.1002/ece3.3322

**Published:** 2017-08-28

**Authors:** Ladd Irvine, Daniel M. Palacios, Jorge Urbán, Bruce Mate

**Affiliations:** ^1^ Marine Mammal Institute, Hatfield Marine Science Center, Oregon State University Newport OR USA; ^2^ Departamento de Ciencias Marinas y Costeras Universidad Autónoma de Baja California Sur La Paz Baja California Sur México

**Keywords:** archival whale tags, benthic diving, bio‐logging, dive classification, diving behavior, sperm whale

## Abstract

Here, we describe the diving behavior of sperm whales (*Physeter macrocephalus*) using the Advanced Dive Behavior (ADB) tag, which records depth data at 1‐Hz resolution and GPS‐quality locations for over 1 month, before releasing from the whale for recovery. A total of 27 ADB tags were deployed on sperm whales in the central Gulf of California, Mexico, during spring 2007 and 2008, of which 10 were recovered for data download. Tracking durations of all tags ranged from 0 to 34.5 days (median = 2.3 days), and 0.6 to 26.6 days (median = 5.0 days) for recovered tags. Recovered tags recorded a median of 50.8 GPS‐quality locations and 42.6 dives per day. Dive summary metrics were generated for archived dives and were subsequently classified into six categories using hierarchical cluster analysis. A mean of 77% of archived dives per individual were one of four dive categories with median Maximum Dive Depth >290 m (V‐shaped, Mid‐water, Benthic, or Variable), likely associated with foraging. Median Maximum Dive Depth was <30 m for the other two categories (Short‐ and Long‐duration shallow dives), likely representing socializing or resting behavior. Most tagged whales remained near the tagging area during the tracking period, but one moved north of Isla Tiburón, where it appeared to regularly dive to, and travel along the seafloor. Three whales were tagged on the same day in 2007 and subsequently traveled in close proximity (<1 km) for 2 days. During this period, the depth and timing of their dives were not coordinated, suggesting they were foraging on a vertically heterogeneous prey field. The multiweek dive records produced by ADB tags enabled us to generate a robust characterization of the diving behavior, activity budget, and individual variation for an important predator of the mesopelagos over temporal and spatial scales not previously possible.

## 1 BACKGROUND

Sperm whales (*Physeter macrocephalus*) are a cosmopolitan species found in deep‐water areas (>1,000 m) of all ocean basins (Jefferson, Webber, & Pitman, 2008; Reeves, Stewart, Clapham, & Powell, 2002). Capable of making regular dives over 60 min in duration and to depths in excess of 1,000 m (Amano & Yoshioka, 2003; Aoki et al., 2007; Watwood, Miller, Johnson, Madsen, & Tyack, 2006), they are highly specialized to utilize deep environments that are not accessible to other more shallow‐diving cetaceans. While they feed mainly on cephalopods, sperm whales are generalist foragers, with a composition of prey items that varies regionally (Clarke, 1980; Kawakami, 1980) and that includes fish in some areas (Clarke, Martins, & Pascoe, 1993; Evans & Hindell, 2004; Rice, 1989). Because of the extreme vertical range they cover, sperm whale dives can be divided into shallow (<200–350 m) and deep dives, with foraging typically occurring during deep dives (Aoki et al., 2012; Miller, Johnson, & Tyack, 2004; Watwood et al., 2006). Sperm whales are able to search different portions of the water column by changing the interpulse interval of their echolocation clicks (Fais et al., 2015), allowing them to conserve energy by only making deep dives when prey is available. At short range, a series of rapid clicks called “buzzes” made by the whale are indicative of the whale precisely locating a prey item immediately prior to a capture attempt (Miller et al., 2004). Sperm whales typically spend about 75% of their time foraging (Whitehead, 2003) and over half of their dive cycle detecting and actively capturing prey (Watwood et al., 2006).

Sperm whales in different global regions are socially organized in “clans” that have their own recognizable vocalizations, suggesting they have common behavioral traits that vary regionally (Rendell & Whitehead, 2003; Whitehead, 2003). Within clans, sperm whales form complex matrilineal groups in which older females are found with several generations of their female offspring (Ortega Ortiz, Engelhaupt, Winsor, Mate, & Hoelzel, 2012; Whitehead, 2003). Juvenile males stay with the female groups until they approach sexual maturity, when they form separate bachelor groups. Adult males eventually become more solitary and roam more widely than the female/juvenile groups (Whitehead, 2003). Factors like prey availability and predation risk are thought to influence the size of matrilineal groups in different parts of the world (Gero, Bøttcher, Whitehead, & Madsen, 2016; Jaquet & Gendron, 2009; Whitehead et al., 2012). Groups appear to employ similar foraging methods to locate a range of different prey species according to the local density and distribution (Watwood et al., 2006).

The Gulf of California is a highly productive semi‐enclosed sea (Santamaría‐Del‐Angel, Alvarez‐Borrego, & Müller‐Karger, 1994) that lies between the Baja California Peninsula and mainland Mexico. It is characterized by a narrow shelf and a steep bottom topography that extends to depths >2,000 m. Humboldt squid (*Dosidicus gigas*) occur there in very large numbers (Morales‐Bojorquez, Hernandez‐Herrera, Nevarez‐Martinez, & Díaz‐Uribe, 2012) and are large in size, making them an important prey item for top predators, including fishermen (Markaida & Hochberg, 2005). Sperm whales are present in the Gulf of California year‐round (Jaquet & Gendron, 2002), and forage extensively on Humboldt squid (Davis et al., 2007). They form social groups of similar size to sperm whales in other highly productive parts of the eastern Pacific Ocean like the Galápagos Islands and off northern Chile, although groups in the Gulf of California appear to be more stable, staying together for periods of months (Jaquet & Gendron, 2009).

To date, most efforts to describe diving behavior in sperm whales have been made using short‐duration (<24 hr) data loggers (Aoki et al., 2007; Miller et al., 2004; Teloni, Mark, Patrick, & Peter, 2008; Watwood et al., 2006). A few studies have used longer‐duration (up to 9 days) records involving either a very limited number of individuals (Amano & Yoshioka, 2003) or abstracted data like histogram summaries of dive depth and duration in order to pass through the very limited bandwidth of Argos satellites (Davis et al., 2007). Thus, information related to how sperm whale diving behavior changes over time and space remains limited, and there has not been a rigorous characterization of the dominant dive types or the proportion of time and effort allocated to each. The development of the Advanced Dive Behavior (ADB) tag, a data logger for large cetaceans capable of staying attached for intermediate time periods (weeks to >1 month with a sampling resolution of 1 Hz), has enabled us to obtain detailed dive records from several whale species, including sperm whales (Mate, Irvine, & Palacios, 2016). Here, we present spatially explicit dive data for sperm whales from Gulf of California, use cluster analysis to classify dives based on a range of metrics, and create activity budgets to describe the time allocated to each type. These data are a substantial addition to our understanding of sperm whale behavior and may be applicable to sperm whales in other parts of the world, as they have been found to use similar foraging strategies across different regions (Watwood et al., 2006).

## 2 METHODS

**Figure 1:**
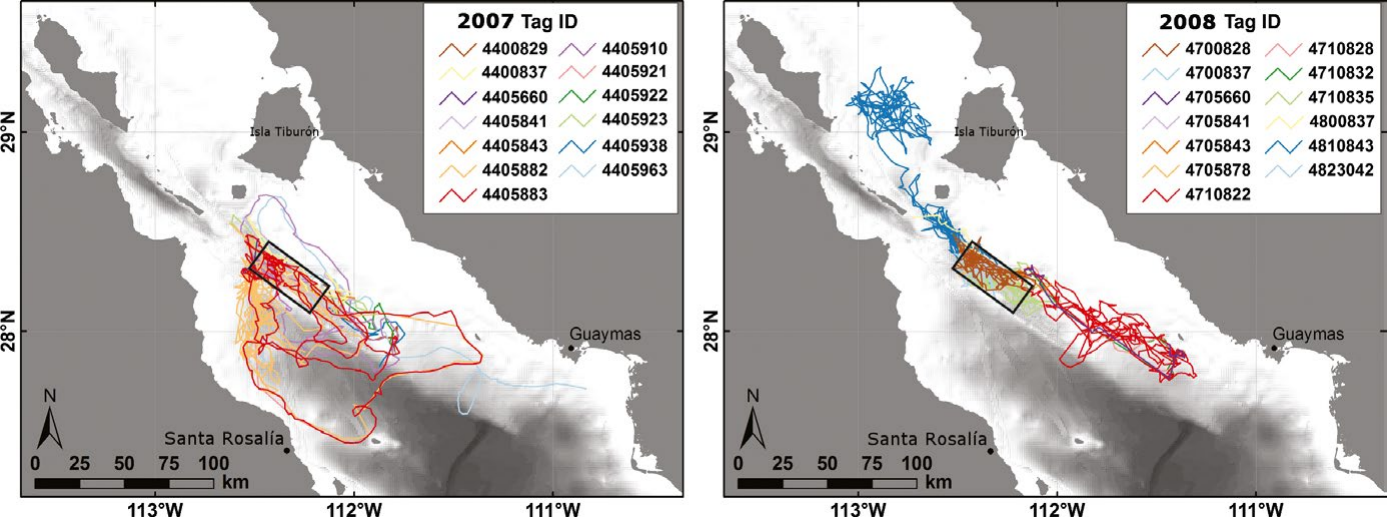
Fastloc GPS locations recorded for sperm whales tracked with Advanced Dive Behavior tags in the central Gulf of California in March–April 2007 (*n* = 13, left) and April & June 2008 (*n* = 13, right. Note: it does not include Tag # 4700827, which did not provide any locations). Tags were deployed within the black rectangle, in the vicinity of Isla San Pedro Mártir

Sperm whales were studied in 2007 and 2008 in the central Gulf of California, Mexico, focusing on whales near Isla San Pedro Mártir (Figure 1). Tagging operations were supported by the 82‐feet‐long R/V *Pacific Storm* during two cruises from 24 March to 15 April 2007 and 20 April to 7 May 2008. A third trip was made on 28 June 2008 using a 50‐feet chartered local vessel.

### 2.1 Tag configuration and deployment

For this study, we used Generation 1 ADB tags, a novel configuration of the Wildlife Computers (Seattle, WA, USA) MK‐10 time‐depth recorder platform, capable of recording depth data at 1‐Hz resolution for multiple weeks before releasing from the whale for recovery. ADB tags also included a Fastloc GPS receiver and patch antenna for acquiring GPS‐quality locations (Bryant, 2007), and an Argos Platform Terminal Transmitter for sending summarized messages via the satellite‐based Argos Data Collection and Location System. Complete details of ADB‐tag construction and design configuration are provided in Mate et al. (2016).

Tags were mounted in a semi‐implantable stainless steel‐housing and deployed at close range (2–4 m) from a 6.8‐m rigid‐hulled inflatable boat using the Air Rocket Transmitter System (ARTS), a modified compressed‐air line‐throwing gun (Heide‐Jørgensen, Kleivane, Øien, Laidre, & Jensen, 2001; Mate, Mesecar, & Lagerquist, 2007). Tags were deployed 0.25–1.5 m forward of the dorsal hump of the whale and no more than 20 cm from the midline, following the protocol described in Mate et al. (2007).

### 2.2 Data collection and transmission

Tags were set to collect depth data at 1 Hz for the duration of the deployment. The data were stored in an onboard archive, and the complete data record could only be accessed by recovering the tag for download. Acquisition of a Fastloc GPS location was attempted immediately after the whale surfaced from dives >10 min in duration and >10 m in depth (“Qualifying Dives”), and a second location was attempted after five minutes if the whale did not descend below 10 m. Pseudoranges for successfully acquired GPS locations were stored in the onboard archive until recovery.

In addition, Argos messages were transmitted continuously every 45 s while the tag was attached to the whale, but were controlled by the saltwater conductivity switch to prevent transmissions while the tag was underwater. An Argos transmission could contain data for one Fastloc GPS location (“Location Message”), summaries of four consecutive Qualifying Dives (“Behavior Message”), or a utility message summarizing battery voltage, number of Argos transmissions, and number of Fastloc attempts. Behavior Messages reported the dive start date/time, maximum dive depth, dive duration, dive shape, and subsequent surface interval duration (post‐dive interval) of four consecutive Qualifying Dives. Tags were programmed to transmit three Location Messages for every one Behavior Message sent due to the larger number of Location Messages that would be generated and fewer locations per message that could be sent compared to dives.

All tags were set to release at 00:00 local time 60 days after the start of field operations and float to the surface for recovery. In the event that the tag and its attachment housing were shed from the whale and sank prior to scheduled release, the tag was programmed to initiate release from its housing after recording a constant depth (±10 m) for 24 hr. Tag release was identified by a change in transmission interval from every 45 s to every 60 s, and new Fastloc GPS locations were subsequently acquired hourly. When a release was identified, tags were located and recovered using a modified uplink receiver that was capable of acquiring, decoding, and solving location messages sent by the tags at a range of 4 km.

### 2.3 Data analysis

Any submergence in the archived depth record lasting longer than 1 min with a maximum depth >10 m was considered a “dive” and isolated for further analysis. Dives were divided into descent, bottom phase, and ascent portions based on the first and last instance where the sign for the rate of change in depth changed or was 0 for 10 consecutive seconds (Aoki et al., 2007). Fastloc GPS locations were assigned to dives that occurred within 2 min of a location. The remaining dives were assigned estimated locations by linear interpolation between the temporally closest Fastloc GPS location before and after the time of the dive. The estimated position of the dive on the interpolated line was determined from the time of the dive relative to the time of the two locations. Dives that occurred more than one hour from the temporally closest location were not included in analyses related to speed or location due to the reduced likelihood of the estimated position reflecting their true position.

Eleven descriptive variables were calculated related to the depth and duration of each dive, or related to surface movement between dives (Table 1). A number of dives involved brief stops at relatively shallow depths during the descent and/or ascent phase of the dive, causing the bottom phase to include a portion of the descent or ascent phase of the dive. To mitigate this, the depth of the descent end point was calculated and divided by the maximum depth of the dive. A subsequent descent end point was then identified (if possible), and the depth where it occurred was also divided by the maximum depth of the dive. If the second descent end point was deeper than the first, and the proportional difference between the two was <0.68 (the mean minus twice the standard deviation; that is, the second end point was significantly deeper than the first end point), the first end point was labeled a “shoulder” and the second end point was used as the end of the descent phase (Fig. S1). A similar process was used for the ascent phase of the dive. These analyses were performed using Matlab^™^.

**Table 1:** Description of summary variables calculated for each dive >10 m depth and >1‐min duration made by ADB‐tagged sperm whales in the Gulf of California

Variable name	Abbreviation	Description	Transformation applied	Pretransformation range
Max Dive Depth (m)	MaxDepth	Maximum depth of the dive	Square root	10–1501
Dive Duration (min)	DiveDur	Duration of the dive	NA	1.0–77.4
Ascent Rate (m/s)	AscRt	Rate of change in depth during the ascent phase of the dive	Square root	0.002–3.15
Descent Rate (m/s)	DescRt	Rate of change in depth during the descent phase of the dive	Square root	0.001–2.57
Bottom Duration (min)	BottDur	Duration of the bottom phase of the dive	Square root	0.02–64.91
Mean Bottom Phase Depth (m)	MeanBottDepth	Mean depth of the bottom phase of the dive	Square root	10.1–1425.5
SD Bottom Phase	SDBottDepth	Standard Deviation of the depth for the bottom phase of the dive	Square root	0.01–311.27
Post‐Dive Interval (min)	PDI	Time between the end of the dive being summarized and the start of the next dive	Log10	0.03–124.42
Bottom Duration/Dive Duration	BotDur/DiveDur	The proportion of the bottom‐phase duration of the dive to the overall dive duration	NA	0.003–0.96
Distance to Seafloor (m)	DistToSeaFlr	The difference between the maximum dive depth and water depth at the dive location	Square root	0–1407
Speed (km/hr)	Speed	Speed of linear travel between consecutive dives (minimum conservative swim speed)	Square root	0.1–10.9
Turning Angle (deg)	TA	The absolute value of the difference in heading between the locations of three sequential dives. The resulting value is reported for the middle of the three dives	Square root	0–179.7

Sperm whales have been known to dive to the seafloor (Heezen, 1957; Mate et al., 2016; Miller et al., 2004; Watwood et al., 2006). To assess the frequency of benthos‐associated diving, the distance from the bottom of a dive to the seafloor depth was included as an additional descriptive variable, for a total of 12 (Table 1). For this purpose, seafloor depth was obtained for each dive location (both true and estimated) from the SRTM‐15 bathymetry dataset (Becker et al., 2009; Smith & Sandwell, 1997), which merges the 1‐arc‐minute resolution ETOPO‐1 altimetry dataset (Amante & Eakins, 2009) with higher‐resolution bathymetry datasets (i.e., multibeam echosounder survey data) where available, into a 15‐arc‐second grid resolution. In several instances, tagged whales were repeatedly recorded diving deeper than the reported seafloor depth nearest to the dive location (*N* = 458), likely due to a combination of uncertainty in the whale's location during the dive, steep bathymetric features, and/or poor‐quality bathymetry data in some areas (Mate et al., 2016). For all such cases, the maximum depth of the dive was assumed to be the seafloor depth for that dive.

Due to large differences in attachment duration between tag deployments, we combined the data from recovered tags to identify dive characteristics common to all tagged individuals. Distributions of the 12 descriptive variables were explored for normality, and relevant transformations were applied (Table 1). Univariate outliers were then identified manually and removed. A principal components analysis (PCA) on the scaled and centered data was subsequently run using the R package “stats” (R Core Team 2016) to reduce dimensionality and to minimize multicollinearity in the data. The combined principal components that explained 85% of the variance were then used as inputs to a hierarchical cluster analysis using the Euclidean distance measure and Ward's method (Legendre & Legendre, 1998) to investigate how dives could be classified into different categories. We note that *k*‐means clustering is a commonly used alternative technique to the hierarchical method (Jain, 2010); however, it is less robust for clusters of unequal size and requires a predetermined number of clusters to be specified (Legendre & Legendre, 1998). In contrast, hierarchical cluster analysis allows the user to select the appropriate number of clusters based on the results by trimming the dendrogram at different levels, allowing for biologically meaningful results to be considered. The stability of cluster analysis results was validated through bootstrapping (1,000 iterations) using the “clusterboot” function from the R package “fpc,” based on the Jaccard similarity value (Henning, 2015).

Activity budgets were constructed for each tagged whale using the dive categories identified by the cluster analysis. The proportion of each dive type made compared to the total number of dives was calculated as well as the proportion of the time spent making dives from each category compared to the overall time spent making dives (i.e., the sum of all Dive Durations and Post‐dive Intervals). The proportion between the sum of the Post‐dive Intervals and the overall time spent making dives was recorded as the percent of time spent at the surface. Locations of each dive type were mapped onto a spatial grid with 5‐km hexagonal cells, and the number of dives falling in each cell was summed to visualize any geographic trends in dive types from the archived data.

We also attempted to classify dives from nonrecovered tags using values from Behavior Messages transmitted through Service Argos. If the Maximum Dive Depth and Duration of a received dive fell within the first and third quartiles of values for the dive types identified from the archived tag data, the dive was assigned to that category; otherwise it was labeled as “Unknown.” The classification of Behavior Message dives from recovered tags was compared to the corresponding dive classifications from the data archive to validate the method. Locations of each dive type were subsequently gridded and mapped, similar to the archived dives for comparison.

Finally, considering that Humboldt squid (an important sperm whale prey item in the Gulf of California) move to shallower depths at night, we investigated possible diel trends for each dive type identified by the cluster analysis using mixed‐effects linear regression. Tag ID was used as a random‐effect grouping variable to control for variability between individuals. Dive Type and Day/Night (Night = 20:00–07:00 local time) indicator variables were used as independent fixed effects with an interaction term. Separate regressions were fit using Maximum dive depth and the number of dives made of each type as response variables.

## 3 RESULTS

A total of 27 ADB tags were deployed in spring 2007 and 2008 near Isla San Pedro Mártir, in the central Gulf of California, Mexico (Figure 1). A total of 10 tags across both years were recovered after having been attached to whales (Table 2; Mate et al., 2016). Overall tracking duration varied from 0 to 34.5 days (median = 2.3 days), and recovered tags were attached for 0.6 to 26.6 days (median = 5.0 days).

**Table 2:** Attachment duration and number of locations from ADB‐tag deployments on sperm whales in the Gulf of California in 2007 (*n* = 13) and 2008 (*n* = 14). Numbers for tags that were not recovered correspond to data collected through Argos summary messages. These numbers differ slightly from those reported in Tables 4 and 5 because those values use the data set that was trimmed of all dives without a location within 60 min (i.e., the data set used in the cluster analysis)

Tag ID #	Deployment date	Duration (days)	# Archived GPS locations	# Transmitted GPS locations	# Archived dives	# Transmitted dives	Recovered?	Estimated length (m)
4405963	27‐Mar‐07	9.7	447	195	362	164	Y^a^, ^d^	9.5
4405841	27‐Mar‐07	2.6	123	38	126	12	Y^b^	9.0
4405843	27‐Mar‐07	0.6	31	20	39	13	Y^b^	9.0
4400837	27‐Mar‐07	2.7	150	45	140	30	Y^b^	8.5
4405938	27‐Mar‐07	1.3	N/A	12	N/A	14	N^a^	8.5
4405923	27‐Mar‐07	2.3	N/A	42	N/A	16	N^a^	14.0
4400829	2‐Apr‐07	0.8	42	25	31	11	Y^b^	11.0
4405883	2‐Apr‐07	13.4	729	211	547	160	Y^b^, ^d^	9.0
4405910	2‐Apr‐07	7.3	439	96	289	46	Y^b^	13.0
4405922	2‐Apr‐07	0.7	38	8	31	0	Y^b^	14.0
4405921	7‐Apr‐07	2.0	N/A	17	N/A	19	N^c^	9.0
4405882	7‐Apr‐07	34.5	N/A	347	N/A	316	N^c^	10.0
4405660	7‐Apr‐07	1.2	N/A	18	N/A	10	N^c^	9.5
4700827	20‐Apr‐08	0.0	N/A	N/A	N/A	N/A	N^a^	14.0
4700828	20‐Apr‐08	11.2	N/A	200	N/A	166	N^a^	8.0
4700837	20‐Apr‐08	9.5	281	249	348	226	Y	9.5
4705841	24‐Apr‐08	1.5	N/A	20	N/A	26	N^a^	10.0
4705878	24‐Apr‐08	1.4	N/A	8	N/A	2	N^a^	10.5
4710835	24‐Apr‐08	17.8	N/A	188	N/A	157	N^a^	9.5
4705660	29‐Apr‐08	1.6	N/A	23	N/A	16	N^a^	11.5
4705843	29‐Apr‐08	0.5	N/A	9	N/A	9	N^a^	9.0
4710822	29‐Apr‐08	15.5	N/A	293	N/A	259	N^a^	10.0
4710828	29‐Apr‐08	2.5	N/A	49	N/A	22	N^a^	9.5
4710832	29‐Apr‐08	2.2	N/A	32	N/A	16	N^a^	9.0
4800837	28‐Jun‐08	13.3	N/A	52	N/A	36	N^a^	9.0
4823042	28‐Jun‐08	1.3	N/A	18	N/A	14	N^a^	8.5
4810843	28‐Jun‐08	26.6	850	309	1,183	251	Y	11.0
	Median	2.3	215.5	40.0	214.5	20.5		

a Stopped Transmitting.

b Released Prematurely.

c Fixed to housing. No recovery possible.

d Found on the beach and returned.

Recovered tags archived a median of 50.8 Fastloc GPS locations per day (range = 29.6–60.1; Table 2) and a median of 33.6% of those locations were received through Service Argos (Table S1). Archived dive profiles recorded a median of 42.6 Qualifying Dives per day (range = 36.6–51.9). Summaries for 0–316 Qualifying Dives were transmitted through Argos as Behavior Messages by all tags.

**Figure 2:**
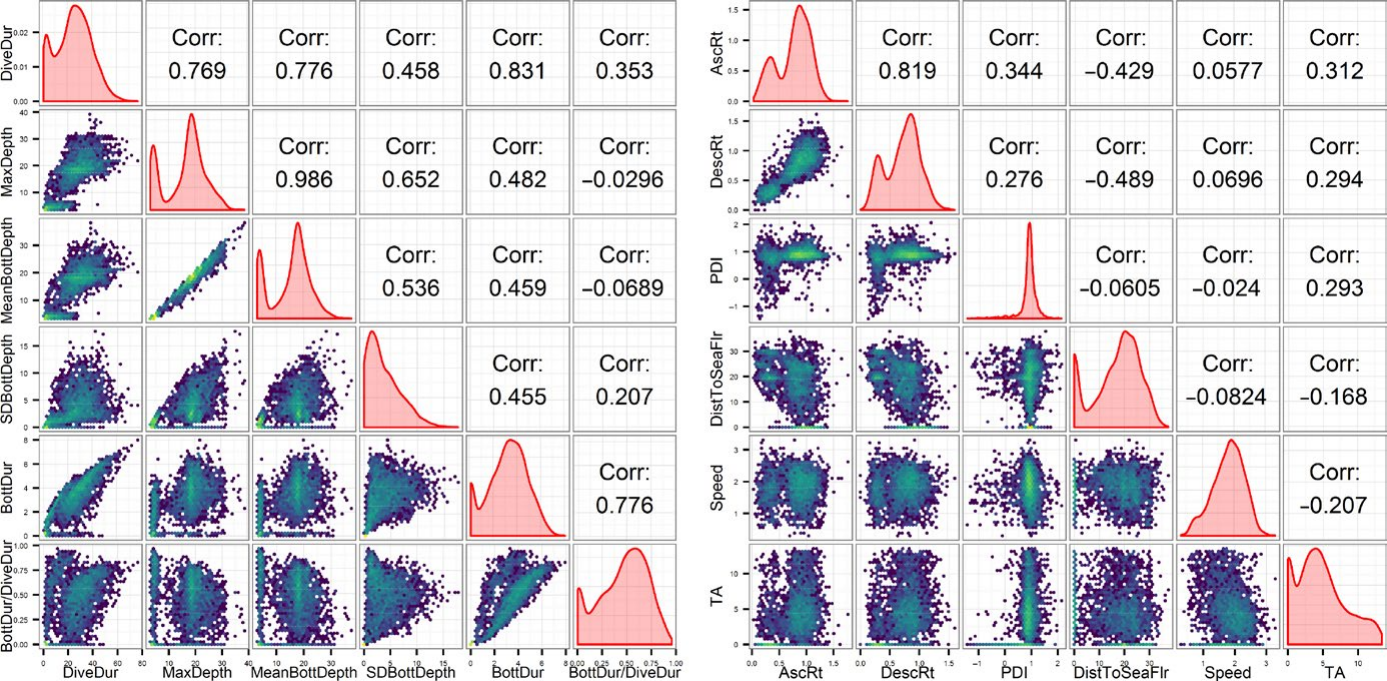
The distributions of 12 variables used in a hierarchical cluster analysis to categorize sperm whale dives after having been transformed as described in Table 1. Univariate kernel density distributions of each variable are shown in red on the diagonal. The correlation matrix is displayed above the diagonal, and the hexagonal binned plots below the diagonal represent the density of points in a scatter plot comparing the two listed variables. Lighter colors represent a higher density of points (range 1–269), and the results were log‐transformed to better visualize density differences. For better visualization, the variables were separated into two plots of six variables each, with depth and duration variables in one plot and the rest of the variables in the other

Tagged whales generally remained near the tagging area at the edge of a deep submarine canyon, with occasional excursions into shallower water (Figure 1). The distribution of archived Maximum Dive Depths for recovered tags was bimodal (Figure 2) with peaks at <50 m and from 300 to 500 m. The mean Maximum Dive Depth across all tags was 325 m (*SD* = 239) with occasional dives as deep as 1,500 m. The mean Dive Duration was 25.4 min (*SD* = 14.2), though one whale (# 4810843) made dives up to 75 min. The interquartile range of Maximum Dive Depth differed substantially across individuals, with some whales diving to a range of depths over twice as wide as others (range = 198–418 m). One whale (# 4405910 in 2007), when diving deeper than 30 m, dived almost exclusively to a depth of 340 ± 20 m regardless of the duration of the dive, time of day, or location.

**Figure 3:**
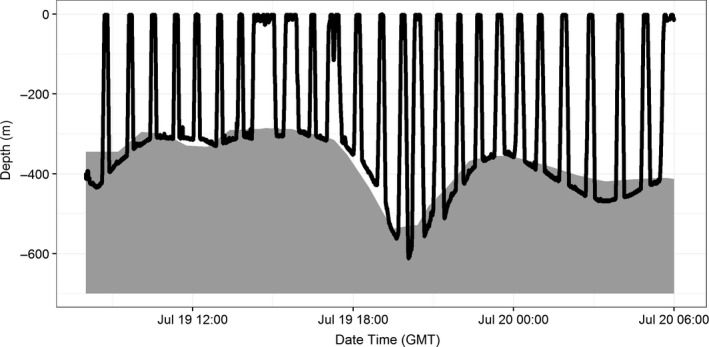
A 17‐hr portion of dive profile from Advanced Dive Behavior Tag # 4810843 attached to a sperm whale in the Gulf of California in June 2008 (black line). The gray polygon shows the depth of the seafloor (from SRTM) nearest to the Fastloc GPS location collected by the tag after the whale surfaced from the dives. The dive profile appears to show the whale following the contour of the bottom during 17 consecutive deep dives

Whale # 4810843 in 2008 was tracked for the longest duration of all recovered tags (26.6 days) and was the only one to travel substantially northward after tagging. After remaining in the tagging area for 13 days, the whale moved to an area north of Isla Tiburón for the remainder of the tracking period (Figure 1). When the whale moved to this new area, its diving behavior changed. The bottom portion of the whale's deep dives had very little vertical variability and instead changed depth gradually, with the bottom of a subsequent dive beginning close to the depth where the prior dive had stopped. Graphical inspection of the depth profiles indicated that the whale appeared to be following the seafloor (Figure 3).

A total of 2,872 archived dives from recovered tags were included in the PCA after removal of outliers (*n* = 1 from Post‐dive Interval, and *n* = 3 from Speed) and dives that occurred more than 60 min from a Fastloc GPS location (*n* = 32). The results indicated that 85% of the variability in the recorded diving behavior could be explained by the first five principal components (Tables 3 and S2). Distance to Sea Floor was the only positive loading for Component 1 and was inversely related to Dive Duration, Maximum Dive Depth, Mean Bottom Phase Depth, SD Bottom Phase Depth, Bottom Phase Duration, Ascent Rate, and Descent Rate (Table 3). Bottom Phase Duration and BottDur/DiveDur were the dominant loadings in Component 2, and were inversely related to MaxDepth and Mean Bottom Phase Depth. Speed was the strongest loading in Component 3 and had a strong inverse relationship with Turning Angle, and to a lesser extent with Post‐dive Interval and Distance to Sea Floor. Post‐dive Interval and Speed were the two strongest loadings in Component 4 and were only weakly opposed by SD Bottom Phase Depth. SD Bottom Phase Depth and Distance to Sea Floor were the two strongest loadings of Component 5 and were inversely related to Dive Duration (Table 3).

**Table 3:** Principal components analysis loadings on the 12 behavior‐related variables for the five components that explained 85% of the variance in tagged sperm whale diving behavior, and their accompanying interpretations

	Dive Duration	Max Dive Depth	Mean Bottom Depth	SD Bottom Depth	Bottom Duration	Ascent Rate	Descent Rate	Post‐Dive Interval	BottDur/TotalDur	Dist to Bottom	Speed	Turning Angle
PC1	−0.365	−0.39	−0.382	−0.277	−0.304	−0.355	−0.353	−0.187	−0.11	0.256	−0.011	−0.189
PC2	−0.174	0.236	0.26	−0.018	−0.507	0.206	0.169	−0.013	−0.713	0.017	0.044	−0.086
PC3	0.01	0.006	0.012	−0.01	0.073	0.032	0.095	−0.355	0.091	−0.287	0.695	−0.532
PC4	−0.013	0.03	−0.002	0.155	−0.019	−0.045	0.05	−0.736	−0.008	−0.383	−0.531	0.004
PC5	0.225	−0.079	0.063	−0.775	0.021	−0.046	−0.061	0.148	−0.112	−0.534	−0.058	0.103

Interpretation of PCs: PC1, Contrasting long‐duration, deep dives and short‐duration, shallow dives; PC2, V‐shaped vs. Square‐ or U‐shaped dives; PC3, Traveling vs. foraging/socializing dives; PC4, Shallow dives with long surface intervals, high travel speed and little variability in depth compared to deeper, more variable dives with shorter surface intervals; PC5, Shallower, shorter duration dives with greater variability in bottom‐phase depth.

**Figure 4:**
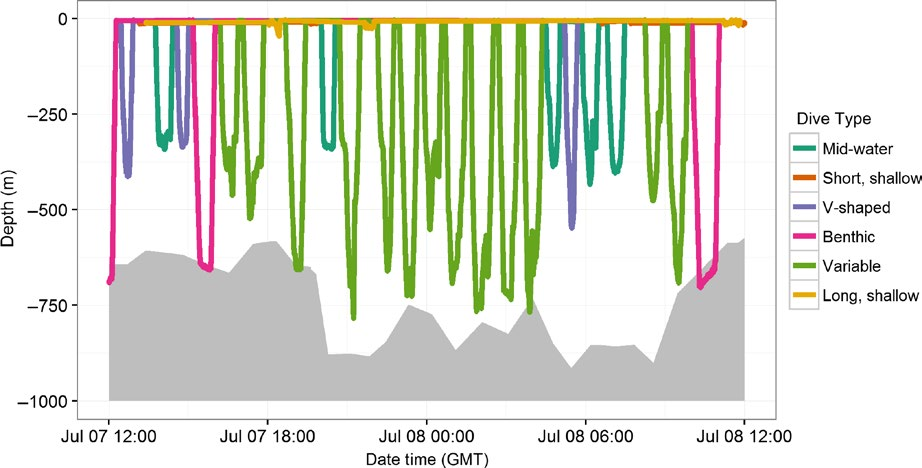
A 24‐hr portion of a sperm whale depth profile (Advanced Dive Behavior Tag # 4810843) from 7 to 8 July 2008 with dives colored to correspond to the type identified by the cluster analysis. The seafloor depth (from SRTM) nearest to each dive location is represented by gray polygon

A cluster analysis using the first five principal components as inputs indicated that dives could be classified into as many as six groups (Fig. S2). The bootstrapping procedure indicated a relatively low degree of stability (Jaccard similarity values <0.6) for several of the six clusters (mean Jaccard similarities = 0.48, 0.56, 0.72, 0.48, 0.53, 0.84, respectively). However, reducing the number to five clusters only improved the stability of one cluster (mean Jaccard similarities = 0.65, 0.53, 0.71, 0.50, 0.83, respectively). Clustering with alternative distance measures (Jaccard and Bray–Curtis) produced similar clustering results but with inferior validation metrics, so the dives were classified into six types using the Euclidean distance method (Table 4, Figure 4), resulting in two “shallow” dive types and four “deep” dive types. “Shallow, short‐duration” dives were characterized by shallow Max Dive Depths (median = 16 m) and short Dive Durations (median = 2.3 min) with very short Bottom Phase Duration. “Shallow, long‐duration” dives were shallow dives (median = 24.4 m) that had a high proportion of the bottom phase to overall dive duration (median = 0.7) and were intermediate in duration (median = 11.0 min). Both shallow dive types had slow Ascent and Descent Rates (median = 0.1 m/s for both). “V‐shaped” dives were intermediate‐duration (median = 21.4 min) deep dives (median = 290 m) whose bottom phase was a very small to nonexistent proportion of the overall dive duration (median = 0.2). “Mid‐water” dives were deep, long‐duration dives (median = 340 m and 30.3 min, respectively), with a relatively high proportion of the bottom‐phase duration to the overall duration (median = 0.5) of somewhat variable depth (median *SD* Bottom Phase Depth = 19.4 m) that did not come close to the sea floor. “Benthic” dives were the longest duration dives (median = 45.8 min) and almost always reached the seafloor (median Distance to the Sea Floor = 0.0 m). There was very little variability in the depth of the bottom phase of the dive (median *SD* Bottom Phase Depth = 6.9 m) and they were also characterized by a very high proportion of the bottom‐phase duration to the total dive duration (median = 0.7). The last category was “Variable” dives, which appeared to be a combination of the other deep dive types, as their distinguishing characteristic was a highly variable depth during the bottom phase of the dive (median *SD* Bottom Phase Depth = 60.3 m). All four of the “deep” dive types were characterized by rapid ascent and descent phases, though V‐shaped dives had the slowest ascent and descents of the group.

**Table 4:** Summary of cluster analysis results, where dives were clustered based on 12 behavior‐related variables. Values shown are median and range for each variable, and classified into one of six dive types (rows)

	*N*	Dive Duration (min)	Max Dive Depth (m)	Mean Bottom Depth (m)	SD Bottom Depth	Ascent Rate (m/s)	Descent Rate (m/s)	Bottom Duration (min)	Post‐Dive Interval (min)	BottDur/TotalDur	Dist to Bottom (m)	Speed (km/hr)	Turning Angle (deg)
Mid‐water	894	30.3 (5.8–61.2)	340.0 (119.2–581.2)	310.2 (74.2–549.1)	19.4 (0.7–165.9)	0.8 (0.1–1.9)	0.7 (0.0–1.8)	16.0 (1.0–49.9)	8.4 (1.1–124.4)	0.5 (0.1–0.9)	388.9 (0.0–1406.8)	3.8 (0.1–8.7)	22.9 (0.0–178.9)
Short, shallow	313	2.3 (1.0–24.1)	16.0 (10.4–310.8)	15.0 (10.2–305.8)	0.0 (0.0–15.3)	0.1 (0.0–2.2)	0.1 (0.0–1.4)	0.0 (0.0–10.2)	3.9 (0.0–92.0)	0.0 (0.0–0.8)	547.1 (115.0–1195.6)	3.2 (0.3–10.7)	0.0 (0.0–125.9)
V‐shaped	490	21.4 (1.3–48.1)	290.0 (42.6–832.0)	281.5 (15.4–832.0)	5.9 (0.0–101.9)	0.6 (0.1–3.2)	0.5 (0.1–2.2)	4.2 (0.0–18.3)	7.5 (0.1–49.3)	0.2 (0.0–0.9)	458.3 (0.0–1195.0)	3.5 (0.1–9.1)	17.5 (0.0–177.8)
Benthic	268	45.8 (27.3–77.3)	456.5 (203.0–978.2)	442.0 (198.7–973.0)	6.9 (0.8–128.4)	1.0 (0.6–1.7)	1.0 (0.3–1.8)	31.0 (9.5–64.9)	7.8 (2.8–17.9)	0.7 (0.2–0.8)	0.0 (0.0–129.0)	3.2 (0.3–8.2)	30.0 (0.0–178.6)
Variable	520	33.1 (12.1–61.6)	635.0 (267.2–1501.0)	512.6 (154.0–1425.5)	60.3 (0.0–311.3)	1.0 (0.1–2.3)	0.9 (0.3–2.6)	14.2 (0.0–37.9)	8.0 (4.0–49.3)	0.5 (0.0–0.8)	80.1 (0.0–943.0)	3.6 (0.2–10.9)	25.3 (0.0–179.7)
Long, shallow	387	11.0 (1.3–44.9)	21.4 (10.6–206.2)	17.0 (10.1–122.1)	1.8 (0.1–66.9)	0.1 (0.0–1.1)	0.1 (0.0–0.5)	8.0 (0.7–39.9)	6.0 (0.2–81.5)	0.7 (0.2–1.0)	434.8 (127.2–1113.0)	3.0 (0.3–9.2)	8.0 (0.0–173.1)

The mixed‐effects linear regression indicated that there were no significant diel differences in the number of dives made of any type (*p* = .696 for Dive Type‐Day/Night interaction term). However, the predicted Maximum Dive Depth of Shallow, short‐duration dives was 9.6 m deeper at night (*p* = .0243), and there was weak evidence for a similar trend in Benthic dives (*p* = .079). No further evidence of diel variability in Maximum Dive Depth was found for other dive types (*p* > .18 for all other dive types).

While there was substantial variability between individuals in the proportion of archived dive types recorded, a median of 74% of the dives were deep dives (Table 5). Mid‐water dives were generally the most common dive type (median = 33.0%) followed by Variable dives (median = 22.3%) and V‐shaped dives (median = 18.0%). Benthic dives were the least common dive type (median = 0.9%) with the exception of whale # 4810843 in 2008, which made a substantially larger percentage of benthic dives (21.9%) than any other tagged whale, and of whale # 4400829 in 2007, which only recorded 27 dives used for the cluster analysis. Benthic dives were recorded by six of the 10 recovered tags. However, almost one‐third of Variable dives also extended to the seafloor, so if we also consider Variable dives, all but one recovered tag (whale # 4405922) recorded dives to the seafloor. Tag archives showed the whales spent a mean of over one‐third of the tracking period on the surface (Table 5), in some cases for well over 30 min at a time. Seven of the 10 recovered tags recorded surface periods (no dives > 10 m deep and > 1‐min duration) in excess of 30 min at least once, and five of the 10 tags recorded at least one surface period lasting over 60 min. One tag (whale # 4700837, tracked for 9.5 days in 2008) recorded a surface period lasting 17.7 hr while moving <4 km. Across all recovered tags, at least one surface duration >30 min was recorded following each dive type except for Benthic dives.

**Table 5:** Activity budget for ten sperm whales tracked in the Gulf of California, showing the percentage of each dive type made by each whale, as well as the percentage of the overall tracking period used to make each dive type. The means are also indicated

Tag ID #	*N* dives/Total time (day)	Mid‐water	Short, shallow	V‐shaped	Benthic	Variable	Long, shallow	Proportion of surface time
4400829								
Dives	27	55.6	7.4	14.8	18.5	3.7	0.0	N/A
Time	0.8	52.1	0.3	3.3	18.6	3.3	0.0	22.3
4400837								
Dives	130	29.2	14.6	26.2	0.0	21.5	8.5	N/A
Time	2.7	25.9	1.0	19.2	0.0	18.6	3.4	32.0
4405841								
Dives	123	35.0	8.9	14.6	1.6	26.0	13.8	N/A
Time	2.6	31.2	1.1	8.0	1.8	27.1	5.1	25.8
4405843								
Dives	26	23.1	15.4	26.9	3.8	23.1	7.7	N/A
Time	0.6	26.1	1.2	15.8	5.0	23.8	1.4	26.7
4405883								
Dives	492	40.2	3.3	21.1	0.2	32.9	2.2	N/A
Time	13.4	32.7	0.7	12.5	0.3	29.3	1.2	23.3
4405910								
Dives	282	63.5	13.8	11.7	0.0	0.7	10.3	N/A
Time	7.3	50.9	0.9	6.0	0.0	0.6	3.9	37.7
4405922								
Dives	29	31.0	17.2	3.4	0.0	0.0	48.3	N/A
Time	0.7	38.5	1.7	0.7	0.0	0.0	19.5	39.7
4405963								
Dives	310	35.8	4.2	27.4	2.3	26.5	3.9	N/A
Time	9.7	30.2	0.4	15.6	2.7	24.9	1.0	25.2
4700837								
Dives	301	24.6	3.7	29.9	0.0	34.6	7.3	N/A
Time	9.5	19.3	0.5	20.2	0.0	30.1	2.1	27.8
4810843								
Dives	1,152	19.2	16.8	9.9	21.9	8.9	23.4	N/A
Time	26.6	20.5	1.6	6.0	31.0	10.7	8.2	22.0
Median								
Dives	206	33.0	11.4	18.0	0.9	22.3	8.1	N/A
Time	5.0	30.7	1.0	10.2	1.0	21.2	2.7	26.2

**Figure 5:**
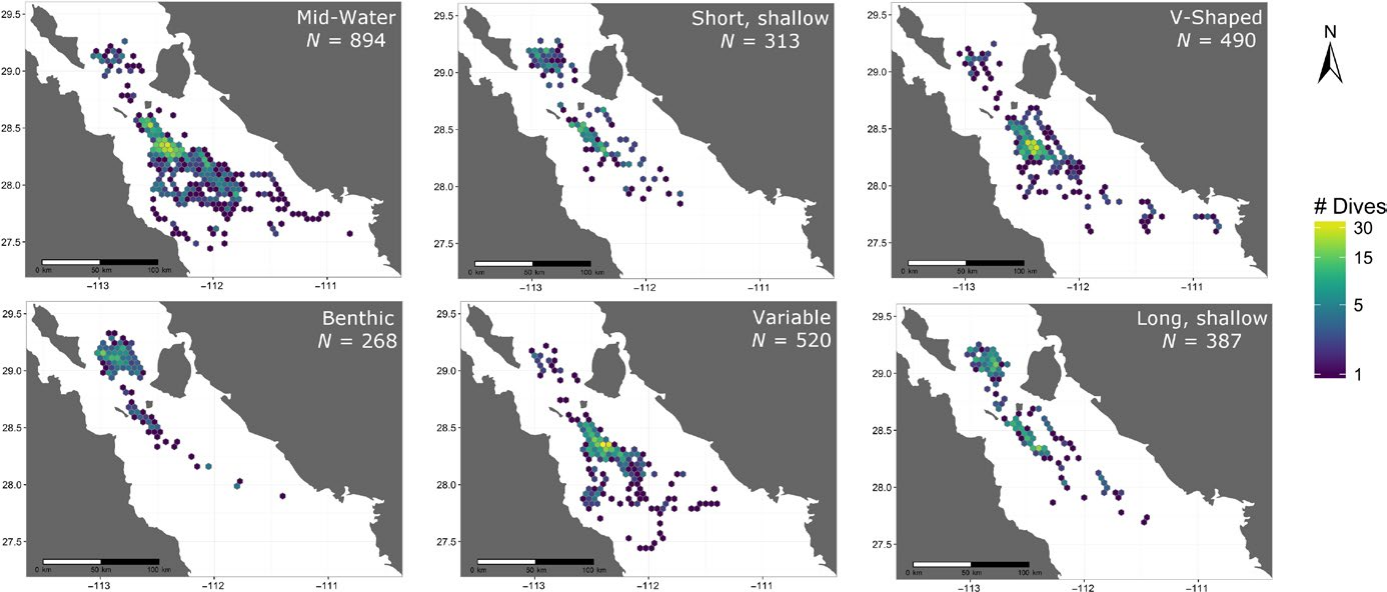
Density of occurrence for each of six dive types derived from recovered Advanced Dive Behavior tags deployed on sperm whales in the Gulf of California during spring 2007 and 2008. A 5‐km grid was used, and lighter colors represent a higher density of dives in that cell (range 1–38). Results were log‐transformed to better visualize spatial variability of dive density

Mid‐water dives occurred across the majority of the study area, with V‐shaped and Variable dives also distributed across the area, although less so at the outer portions (Figure 5). All three dive types occurred most frequently near the tagging area where the highest overall number of dives was recorded. Benthic dives were the dominant dive type made by whale # 4810843 in the area north of Isla Tiburón in 2008 (Figure 5). They were also recorded near the tagging area by other tags; however, they occurred much less frequently there, and rarely were made in multiple sequential dives. Two whales in 2007 (# 4405910 and # 4405963) made separate circuits north of the tagging area in the same week (Figure 1), with one whale making almost exclusively “shallow” dives, while the other made almost exclusively V‐shaped dives during the trips.

Argos Behavior Message transmissions summarized 2017 dives from all tags (recovered and nonrecovered). A total of 913 of those summarized dives were from recovered tags and 584 of them could be classified using the archived dive type quantiles to validate the classification of summarized dives from nonrecovered tags. No received dives were classified as Shallow, short‐duration dives, and relatively few were classified as Shallow, long‐duration dives due to the programmed criteria that only dives >10 m depth and >10‐min duration would be summarized by Behavior Messages. Over 77% of summarized dives from recovered tags that were classified as either Benthic or Variable dive types were correctly attributed when compared to the archived dive classification (Table S3). Correct classification of V‐shaped and Mid‐water dives occurred 52% and 64% of the time, respectively. Misclassifications of Mid‐water dives were most often labeled as V‐shaped and vice versa.

A total of 1,103 summarized dives were received from nonrecovered tags and 787 of those were able to be classified. V‐shaped dives were the most common type for nonrecovered dives (*n* = 359), followed by Mid‐water (*n* = 249) and Variable (*n* = 155) types. Only one dive was classified as Benthic and 23 were classified as Shallow, long dives. The three most dominant dive types were all abundant near the tagging area, similar to what was observed from the archived tags, with Mid‐water and V‐shaped dives also occurring south toward Santa Rosalía and southeast toward Guaymas (Fig. S3).

**Figure 6:**
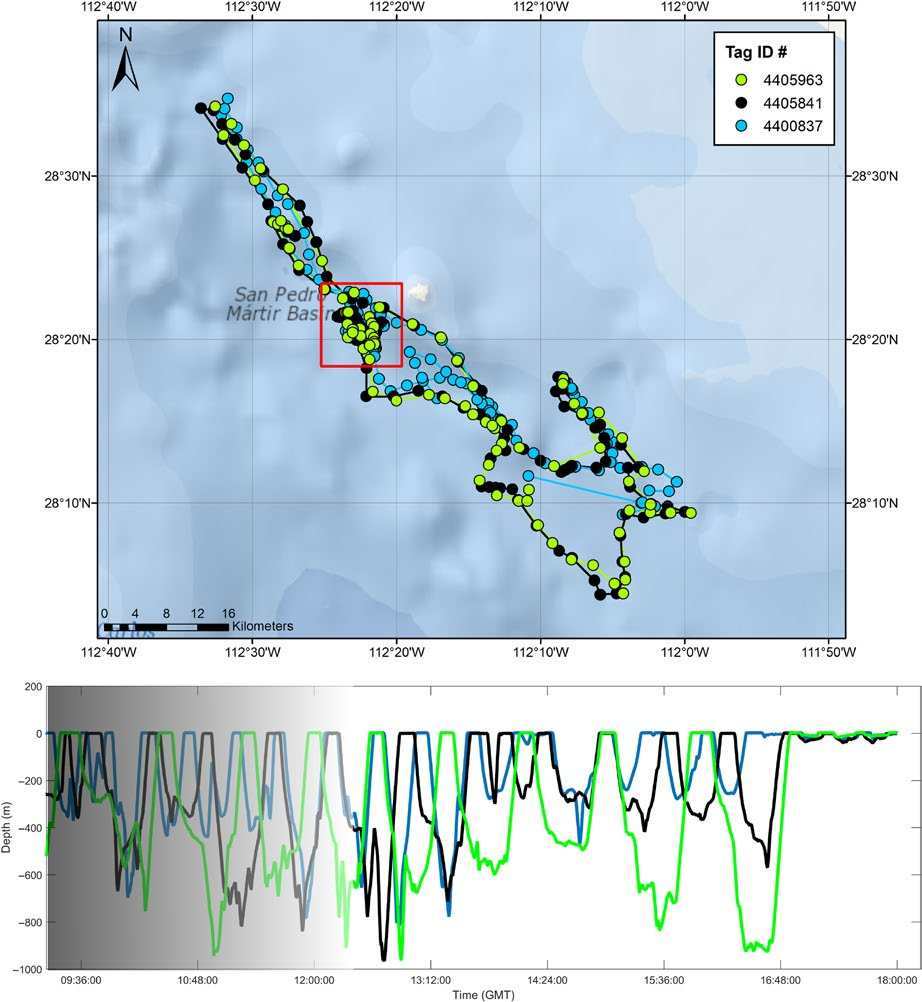
A 2.5‐day portion of Fastloc GPS tracks for three sperm whales tagged with Advanced Dive Behavior tags on the same day (27 March 2007) moving in close proximity to each other (Upper panel). Dive profiles from the same three whales while they were moving in close proximity, showing asynchronous diving behavior in both time and depth (Lower Panel). Dive profiles are from the area highlighted by the red box in the upper panel, corresponding to the time period 09:00–18:00 GMT. The highlighted area was chosen due to the cluster of locations, which would suggest the whales were most likely to be foraging. An approximately equal number of each dive type except Benthic (*n* = 0) was recorded in the highlighted area for Tags 4400837 and 4405841, but no dives from tag 4405963 were recorded as Mid‐water or V‐shaped

In 2007, three whales (# 4400837, # 44005841, # 4405963) were tagged on the same day and were recorded traveling in close proximity (<1 km) to each other for the next 2 days, likely as a part of a social group. All three whales were estimated to be approximately the same size (8.5–9.5 m; Table 1). The whales remained nearby (but not in such close proximity) after that time period for another 0.5 days, until tags # 4400837 and # 44005841 both released prematurely from the whales after having traveled 225 km. While the surface movements were quite synchronous among these three whales, the timing and depth of their dives were mostly not (Figure 6). The whales generally dived at staggered intervals, where no more than two were at the surface at any time and rarely were diving to the same depth range. These whales also made differing numbers of dive types (Table S4), with whale # 4400837 making the most V‐shaped dives and spending the most time at the surface, while the other two were somewhat similar, with one (whale # 4405841) making slightly more Mid‐water dives and the other (whale # 4405963) making more Benthic and Variable dives.

## 4 DISCUSSION

Despite some excursions, the movements of tagged whales were mostly limited to the northern part of the San Pedro Mártir Basin near the tagging area, in water averaging 720 m deep. This area is reported as a spawning area for Humboldt squid (Gilly, Elliger, et al., 2006), a large cephalopod that is the primary prey source of sperm whales in the area (Davis et al., 2007; Ruiz‐Cooley, Gendron, Aguíñiga, Mesnick, & Carriquiry, 2004). Sperm whales tend to travel linearly when feeding success is low (Whitehead, 2003), so the high density of locations in the area suggests the whales were foraging successfully. The population of Humboldt squid in the Gulf of California has been estimated to be as large as 132 million squid (Morales‐Bojorquez et al., 2012) and they are the target of a commercial fishery located on either side of the Guaymas Basin that harvests as many as 100,000 tons annually. The timing of squid catches and mark–recapture studies show that the squid migrate across the Gulf of California (Gilly, Markaida, et al., 2006; Markaida, Rosenthal, & Gilly, 2005), suggesting they are abundant throughout the study area year‐round. Recruitment appears to peak in late spring (May; Morales‐Bojorquez et al., 2012), further suggesting sperm whale prey was especially abundant in the area when our tags were deployed. Additionally, the movement of tagged whales south and southeast of the tagging area corresponds to two areas (Santa Rosalía and Guaymas) that are known from the fishery to support large concentrations of squid (Gilly, Markaida, et al., 2006; Markaida et al., 2005), so the whales may have been searching for additional prey concentrations there.

Principal components analysis of dive behavior metrics revealed that five components explained 85% of the variability in diving behavior and interpretation of the component loadings (Table 3) was relatively straightforward. PC1 contrasts short, shallow dives with longer‐duration dives to deeper depths. The strong positive correlation between Maximum Dive Depth and Dive Duration and their inverse relationship to Distance to the Bottom represents the extra travel time needed to reach deeper depths and likely the increased foraging time in deeper dives predicted by diving physiology models (Houston & Carbone, 1992; Kramer, 1988). Ascent and Descent Rate were also correlated to Maximum Dive Depth, as it is most efficient to travel rapidly to and from deeper depths in order to maximize the time at depth. PC2 differentiated between dives with little to no bottom phase (V‐shaped) and those with an extended bottom phase (square or U‐shaped). The lesser, but opposite, influence of Maximum Dive Depth and Mean Bottom Phase Depth may relate to distinguishing the Shallow, long dives from deeper dives. PC3 indicated that turning angles and postdive intervals were reduced during higher travel speeds, which would be expected as whales typically travel faster during linear travel segments (Lagerquist, Mate, Ortega‐Ortiz, Winsor, & Urban‐Ramirez, 2008; Mate, Best, Lagerquist, & Winsor, 2011; Mate, Lagerquist, & Calambokidis, 1999). Therefore, PC3 contrasted traveling dives and foraging dives or dives related to socializing where the whales spend more time at the surface and in a smaller area. PC4 was somewhat difficult to interpret in light of PC3, as it indicated that dives with longer postdive intervals had higher travel speeds and were shallower (larger Distance to the Sea Floor). However, the moderate, opposite effect of *SD* of Bottom Phase Depth may indicate that PC4 describes exploratory dives where the whale slowed down to search an area more intensely. Finally, the strong loadings of *SD* of Bottom Phase Depth and Distance to the Sea Floor in PC5 describe highly variable dives and suggest there was more variability in the depth of the bottom phase for Shallow, short‐duration dives.

The accuracy of the Gulf of California bathymetry data may have been a confounding factor for proper dive classification. Many of the instances where maximum dive depth was deeper than the reported seafloor depth were small enough (<50 m; *n* = 283) to be the result of imprecise knowledge of the whale's location during a dive, or of small‐scale variability of the bottom topography not resolved by current bathymetric products. However, the occasional discrepancy of more than 200 m (*n* = 30) suggests there are areas of uncharted seafloor relief in the current bathymetric products. While it is not known if dives exceeding the reported seafloor depth actually reached all the way to the seafloor, the presence of many dives that traveled to the reported seafloor depth, and the shape of those dives that suggests the whale was following the seafloor, gives us confidence in assigning the seafloor depth as equal to the maximum dive depth recorded by the tag for those locations. It is also possible that Mid‐water or Variable dives could be mistaken for Benthic dives in areas where the seafloor is substantially deeper than reported, and the Distance to the Seafloor would therefore be erroneously small. There is no way to know how many of these instances exist in the data; however, as Distance to the Seafloor was only one of 12 metrics used to group dive types, such errors should have a relatively small effect.

The cluster analysis identified six types of dives (Mid‐water, V‐shaped, Benthic, Variable, Shallow, long and Shallow, short); however, the relatively low stability of some of the clusters suggests these are general trends in the diving behavior, but that precise classification of every dive is difficult. The median Maximum Dive Depth of Mid‐water dives (340 m) was shallower than sperm whale foraging dives from other parts of the world in lower latitudes (600–900 m; Aoki et al., 2012; Watwood et al., 2006; Zimmer, Johnson, D'amico, & Tyack, 2003). However, these depths were comparable to those reported for Gulf of California sperm whales from other studies (Davis et al., 2007), as well as to the daytime depths typically occupied by Humboldt squid (Gilly, Markaida, et al., 2006; Stewart, Field, Markaida, & Gilly, 2013), the primary prey of sperm whales in the Gulf of California. Davis et al. (2007) hypothesized that sperm whales preferentially forage on Humboldt squid within the oxygen minimum zone (OMZ), which typically begins at a depth of ~250 m (Gilly, Markaida, et al., 2006; Markaida et al., 2005), to take advantage of the squid's slowed metabolism despite their adaptations to withstand low‐oxygen environments (Gilly, Markaida, et al., 2006; Stewart et al., 2013). Dives classified as Mid‐water dives, with their rapid descent/ascent phases and a long bottom phase of somewhat variable depth, are characteristic of foraging dives documented in sperm whales from other parts of the world (Fais et al., 2015; Miller et al., 2004), so it is likely that Mid‐water dives in this study represent the tagged whales foraging on Humboldt squid in the OMZ of the Gulf of California.

V‐shaped dive types descended to a similar median Maximum Dive Depth as the Mid‐water dives (290 m vs. 340 m); however, they covered a much wider range of depths and were of shorter duration (median = 21.4 min), with a very short, to nonexistent, bottom phase of the dive. They likely represent exploratory dives where the whale descended to foraging depth but then quickly returned to the surface, perhaps due to a lack of detected prey in the area. While it is theoretically possible for sperm whales to acoustically search the entire water column while near the surface, they appear to adjust the rate of their echolocation clicks to focus on discrete prey patches or sections of the water column (Fais et al., 2015), meaning they would dive close to a foraging depth while searching for prey. Further, sperm whales have been shown to use information from prior dives to dictate the depth and range at which they start searching for prey (Fais et al., 2015). The substantial correlation between Maximum Dive Depth of V‐shaped dives and the previous dive (*r* = 0.511), 72% of which were either V‐shaped or Mid‐water, reinforces this idea.

Benthic dive types were recorded in some proportion by six of the recovered tags (0.2–21.9%; Table 5) and represent a sperm whale diving behavior that has received limited attention so far, perhaps because of the short duration of previous archival tag records. The relatively slow, continuous changes along the bottom phase of Benthic dives strongly suggest the whales were following, and likely foraging along, the seafloor. Sperm whales have previously been noted to forage “benthopelagically,” with a dive shape similar to Mid‐water dives, to depths within 200 m of the seafloor (Fais et al., 2015), and other studies have mentioned dives to the benthos (Heezen, 1957; Miller et al., 2004; Watwood et al., 2006). However, while benthic foraging has been hypothesized based on stomach contents (Clarke, 1980; Martin & Clarke, 1986), there has been no previous assessment of the prevalence of this behavior in sperm whales, or indication that they specifically follow the seafloor during the bottom phase of the dive, as has been shown here. Sharp, vertical excursions during the bottom phase of some Benthic dives may indicate pursuit of prey escaping off the bottom (Figure 3).

Benthic dives were most commonly made by whale # 4810843 in the waters north of Isla Tiburón, where the seafloor is shallower and less variable than the tagging area. The whale's occupation of that area is unusual because, globally, sperm whales are most often found in waters >1,000 m deep (Rice, 1989; Whitehead, 2003; but see Teloni et al., 2008). While sperm whales in the Gulf of California have been found in areas with seafloor depths as shallow as 600 m (Jaquet & Gendron, 2002), the mean seafloor depth occupied by whale # 4810843 north of Isla Tiburón was 392 m (*SD* = 50.2 m). With the extended time the whale spent in the area, it is likely the whale was foraging. However, it is unclear why sperm whales are not observed in other productive shallow water parts of the Gulf of California like near Santa Rosalía, where Humboldt squid are known to be abundant, but sperm whales are only observed in the deeper waters nearby (Davis et al., 2007; Gilly, Markaida, et al., 2006; Markaida et al., 2005).

While the primary prey of sperm whales in the Gulf of California is Humboldt squid, and the squid have been documented diving to depths >1,200 m (Stewart et al., 2013), it is unknown if they were the target species during Benthic dives. It is possible that Benthic dives represent a change in foraging strategy to target a benthic organism. Sperm whales are known to prey on fish in some parts of the world (Clarke et al., 1993; Evans & Hindell, 2004; Rice, 1989), and they also predate on deep‐water, bottom‐dwelling fish like sablefish (*Anoplopoma fimbria*) and Patagonian toothfish (*Dissostichus eleginoides*) from fishermen's demersal longline gear (Hucke‐Gaete, Moreno, Arata, & Ctr, 2004; Sigler, Lunsford, Straley, & Liddle, 2008). It is therefore possible the Benthic dives observed here represent the tagged whales targeting demersal fish that reside in the Gulf of California.

Variable dive types likely represent multiple behaviors and/or a combination of other dive types as their distinguishing characteristic was a highly variable depth of the bottom phase of the dive. Sperm whales focus on discrete prey patches or sections of the water column while descending and searching for prey and have been documented making feeding attempts at much shallower depths than the main depth of a dive (Fais et al., 2015). Therefore, at least some of the Variable dives likely represent stops to forage during planned descents to greater depths, or during ascents. The dive “shoulders” identified during the delineation of different phases of the dive (Fig. S1) may represent a similar behavior. Stewart et al. (2013) documented tagged Humboldt squid making rapid descents through the OMZ to depths >1,200 m, which they hypothesized were predator‐avoidance reactions, likely attempting to escape sperm whales. Some Variable dive types included rapid descents from a mid‐water depth followed by a rapid return to shallower waters that may have been a pursuit of prey (Fig. S4). In other cases, the whale remained at depth suggesting the deeper depths were the originally intended goal of the dive, or that it may have been more energetically efficient to continue foraging at that depth for the remainder of the dive if prey were available.

Many studies of sperm whale diving behavior focus on dives deeper than a certain threshold (typically 200–350 m) where foraging generally occurs, and label the remaining dives as “shallow” (Aoki et al., 2012; Miller et al., 2004; Watwood et al., 2006; Zimmer et al., 2003). The two types of shallow dives identified here indicate there may be additional levels of behavioral complexity within shallow dives. Shallow, short‐duration dives may represent socializing behavior, or possibly be part of an oxygen‐recovery strategy after a deep dive. The significant diel difference in Maximum Dive Depth of Shallow, short‐duration dives is not likely to be biologically significant as the difference in predicted depth (9.6 m) was equivalent to the body length of the tagged whales.

Sperm whales have been documented foraging at depths as shallow as 48 m (Fais et al., 2015), so Shallow, long‐duration dives could represent the whales foraging at shallow depths. However, that would most likely occur at night when Humboldt squid come to the surface waters following the diel migration of the deep scattering layer (Gilly, Markaida, et al., 2006). There was no evidence of diel variation in the occurrence of Shallow, long dives, so they are most likely not related to foraging. It is also possible that some Shallow, long dives were “drift‐dives,” where the whale remains motionless in a vertical posture for an extended period of time and is thought to be resting (Miller, Aoki, Rendell, & Amano, 2008). The median maximum depth of Shallow, long dives reported here (21.4 m) is slightly deeper than those reported by Miller et al. (8.6–16.5 m), but, because the ADB tags used in this study did not have accelerometers to measure body orientation, it is unknown if the difference is due to regional variability in the behavior, or the whales engaging in a different type of behavior.

A median of almost 30% of the recovered tag tracking duration was spent at or near the surface (i.e., the time when the whales were at <10 m or dived for <1 min), exceeding what has been reported for sperm whales in some parts of the world (Miller et al., 2008; Watkins et al., 1999) and approximately equivalent to others (Watwood et al., 2006). While resting behavior has been documented in sperm whales in other studies, it only accounted for 7.1% of the tracking duration (Miller et al., 2008), and it occurred deep enough (8.6–16.5 m) that it likely would have qualified as a dive in our analysis. Sperm whales are known to be highly social animals and regularly form social aggregations where they remain at the surface for >20 min continuously (Watkins et al., 1999; Watwood et al., 2006). Watwood et al. (2006) noted that sperm whales in the Ligurian Sea spent less time at the surface than those in the North Atlantic and speculated that this was related to lower levels of observed social behavior. Sperm whales in the Gulf of California form large social groups that are more stable than similarly sized groups found in other regions (Jaquet & Gendron, 2009). Therefore, the extensive time at the surface recorded by the sperm whales in this study may be a reflection of social activity involving the tagged whales.

Matrilineal‐based social groups of sperm whales travel together for months at a time (Whitehead, 2003); however, little is known about whether this social cohesion is also reflected during periods of foraging at depth. The tracks of three ADB‐tagged whales as they traveled together over 2 days offer one of the first insights into the dynamics of sperm whales traveling and foraging as a group. The close proximity of horizontal travel by the tagged whales and the social cohesion of sperm whales in general raise the possibility they may have been foraging cooperatively, as other highly social marine mammals have been documented acting cooperatively to herd and concentrate prey (Benoit‐Bird & Au, 2009; Vaughn, Würsig, & Packard, 2010). However, the data presented here are insufficient to support such a claim, as the sensors on the tags could not distinguish three‐dimensional movements of the whales during a dive. An alternative model is “local enhancement” where animals dispersed over an area use cues provided by other members of the group to locate prey more rapidly, such as birds travelling to locations where they can see other birds feeding (Beauchamp, 2014; Galef & Giraldeau, 2001). The three tagged whales did not appear to follow this pattern, instead consistently remaining in relatively close proximity to each other during both linear and clustered movement segments. The whales did appear to distribute their effort vertically by alternating both the depth and timing of their dives; however, while that might be interpreted as the whales searching for prey at various depths, they did not appear to converge on a single depth range. Many of the dives during this period were Mid‐water (i.e., foraging) dives, so the variable depths of the dives more likely represent the whales individually foraging on prey with a vertically heterogeneous distribution, as has been described for prey of other deep‐diving marine mammals (Benoit‐Bird, Southall, & Moline, 2016). The temporal staggering of the dives may be an indication that the whales were taking turns maintaining contact with a prey school as it moved, although this could also be interpreted as individuals from the group foraging independently.

While the results presented here provide a detailed view of sperm whale diving behavior over a previously unreachable time period, the ability to identify and describe foraging behavior is a key piece that is missing from the data. Foraging behavior has been described well enough in other work (Aoki et al., 2012; Miller et al., 2004) that the purpose of different dive types described here can be broadly assigned. However, the ability to explicitly identify foraging behavior (e.g., from motion and attitude sensors) would allow for a better understanding of the amount of effort expended by the whales foraging, and how that effort varies both spatially and temporally. Future generations of ADB tags will incorporate three‐axis accelerometers and magnetometers in order to better address these questions.

## CONFLICT OF INTEREST

None declared.

## AUTHOR CONTRIBUTIONS

LI participated in the field work, conducted the data analysis, and wrote this manuscript. DP assisted with data analysis and conducted critical review of the manuscript. JU supplied critical permits, participated in the field work, and reviewed the manuscript. BM conceived of the study, led the field work, and participated in the review of the manuscript.
