# Supplementary material for: Sperm whale dive behavior characteristics derived from intermediate‐duration archival tag data
Source: Ecol Evol. 2017 Aug 28;7(19):7822–37. doi: 10.1002/ece3.3322 (PMC5632629; doi:10.1002/ece3.3322)
Supplement: Supplementary file 1 [file ECE3-7-7822-s001.docx]

SUPPLEMENTAL MATERIAL

Supplemental Table S1: Summary of the number of Fastloc GPS locations and dives > 10 m deep and > 10 min duration transmitted through Service Argos by recovered ADB tags attached to sperm whales in the Gulf of California. The percentage of the overall archived data record represented by the transmitted data is also presented as a measure of data throughput via the Argos system.

| Tag ID # | # Transmitted GPS Locations | % of Archived GPS Locations Transmitted | # Transmitted Dives | % of Archived Dives Transmitted |
| --- | --- | --- | --- | --- |
| 4400829 | 25 | 59.5 | 11 | 44.0 |
| 4400837 | 45 | 30.0 | 30 | 24.2 |
| 4405841 | 38 | 30.9 | 12 | 11.2 |
| 4405843 | 20 | 64.5 | 13 | 52.0 |
| 4405883 | 211 | 28.9 | 160 | 30.4 |
| 4405910 | 96 | 21.9 | 46 | 19.2 |
| 4405922 | 8 | 21.1 | 0 | 0.0 |
| 4405963 | 195 | 43.6 | 164 | 46.1 |
| 4700837 | 249 | 88.6 | 226 | 79.9 |
| 4810843 | 309 | 36.4 | 251 | 31.8 |
| Median | 70.5 | 33.6 | 38 | 31.1 |

Supplemental Table S2: Standard deviation and proportion of variance explained by each component of the PCA.

|  | PC1 | PC2 | PC3 | PC4 | PC5 | PC6 | PC7 | PC8 | PC9 | PC10 | PC11 | PC12 |
| --- | --- | --- | --- | --- | --- | --- | --- | --- | --- | --- | --- | --- |
| Standard deviation | 2.391 | 1.289 | 1.106 | 0.954 | 0.825 | 0.806 | 0.713 | 0.615 | 0.412 | 0.289 | 0.125 | 0.055 |
| Proportion of variance | 0.476 | 0.138 | 0.102 | 0.076 | 0.057 | 0.054 | 0.042 | 0.032 | 0.014 | 0.007 | 0.001 | 0.000 |
| Cumulative proportion | 0.476 | 0.615 | 0.717 | 0.793 | 0.849 | 0.903 | 0.946 | 0.977 | 0.992 | 0.998 | 1.000 | 1.000 |

Supplemental Table S3: A confusion matrix comparing dives classified from Argos-transmitted Behavior Message dive summaries to the ‘true’ classification made using archived data. Behavior message dive summaries were assigned a dive type if their reported dive duration and depth fell within the first and third quartiles of a dive type calculated from archived data.

|  |  |  | **Archived Dives (actual)** | | | |  |  |
| --- | --- | --- | --- | --- | --- | --- | --- | --- |
|  |  | Mid-Water | Short, Shallow | V-Shaped | Benthic | Variable | Long, Shallow | Total |
|  | Mid-Water | 126 | 1 | 42 | 15 | 12 | 0 | 196 |
| **Argos Summarized Dives (predicted)** | Short, Shallow | 0 | 0 | 0 | 0 | 0 | 0 | 0 |
|  | V-Shaped | 56 | 2 | 44 | 2 | 2 | 1 | 107 |
|  | Benthic | 7 | 0 | 0 | 40 | 3 | 0 | 50 |
|  | Variable | 5 | 0 | 11 | 28 | 144 | 0 | 188 |
|  | Long, Shallow | 0 | 3 | 0 | 0 | 0 | 40 | 43 |

Supplemental Table S4: Activity budget for three sperm whales tracked in 2007 with ADB tags that travelled in close proximity for two days. The percentage of each dive type made by each whale (Dives = # dive type / # all dive types), as well as the percentage of the overall period in close proximity that was used to make each dive type (Time = duration of dive type/total time in close proximity) are shown along with the overall median values.

| Tag ID # |  | N Dives/ Total Time (d) | Mid-Water | Short, shallow | V-Shaped | Benthic | Variable | Long, shallow | Proportion of Surface Time |
| --- | --- | --- | --- | --- | --- | --- | --- | --- | --- |
| 4400837 | Dive | 130 | 29.2 | 14.6 | 26.2 | 0.0 | 21.5 | 8.5 | N/A |
|  | Time | 2.7 | 25.9 | 1.0 | 19.2 | 0.0 | 18.6 | 3.4 | 32.0 |
| 4405841 | Dive | 123 | 35.0 | 8.9 | 14.6 | 1.6 | 26.0 | 13.8 | N/A |
|  | Time | 2.6 | 31.2 | 1.1 | 8.0 | 1.8 | 27.1 | 5.1 | 25.8 |
| 4405963 | Dive | 88 | 31.8 | 8.0 | 8.0 | 6.8 | 37.5 | 8.0 | N/A |
|  | Time | 9.7 | 26.6 | 0.5 | 4.2 | 7.4 | 33.8 | 2.2 | 25.3 |
| Median | Dive | 123 | 31.8 | 8.9 | 14.6 | 1.6 | 26.0 | 8.5 | N/A |
| Median | Time | 2.7 | 26.6 | 1.0 | 8.0 | 1.8 | 27.1 | 3.4 | 25.8 |


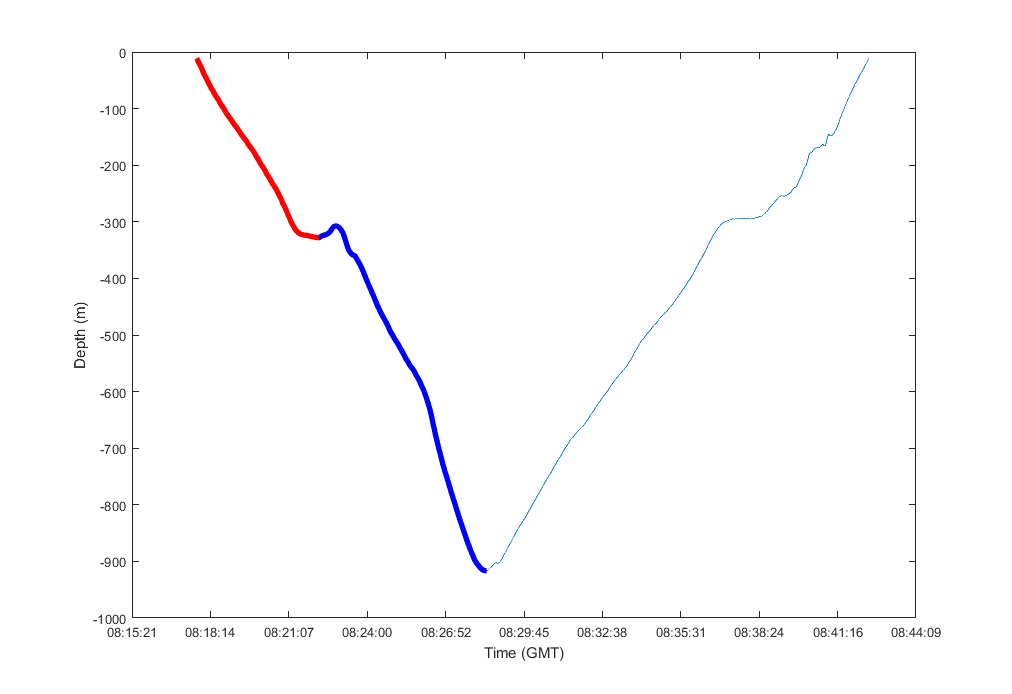


Supplemental Figure S1: An example depth profile from an Advanced Dive Behavior tagged sperm whale showing a ‘dive shoulder,’ where the whale briefly pauses its descent, before continuing deeper in the water column. The thick red portion shows the descent phase of the dive to the first point where the descent rate was 0 or positive for 10 s. The thick red and blue lines together show the new descent phase if the first endpoint of the descent was identified as a ‘shoulder.’


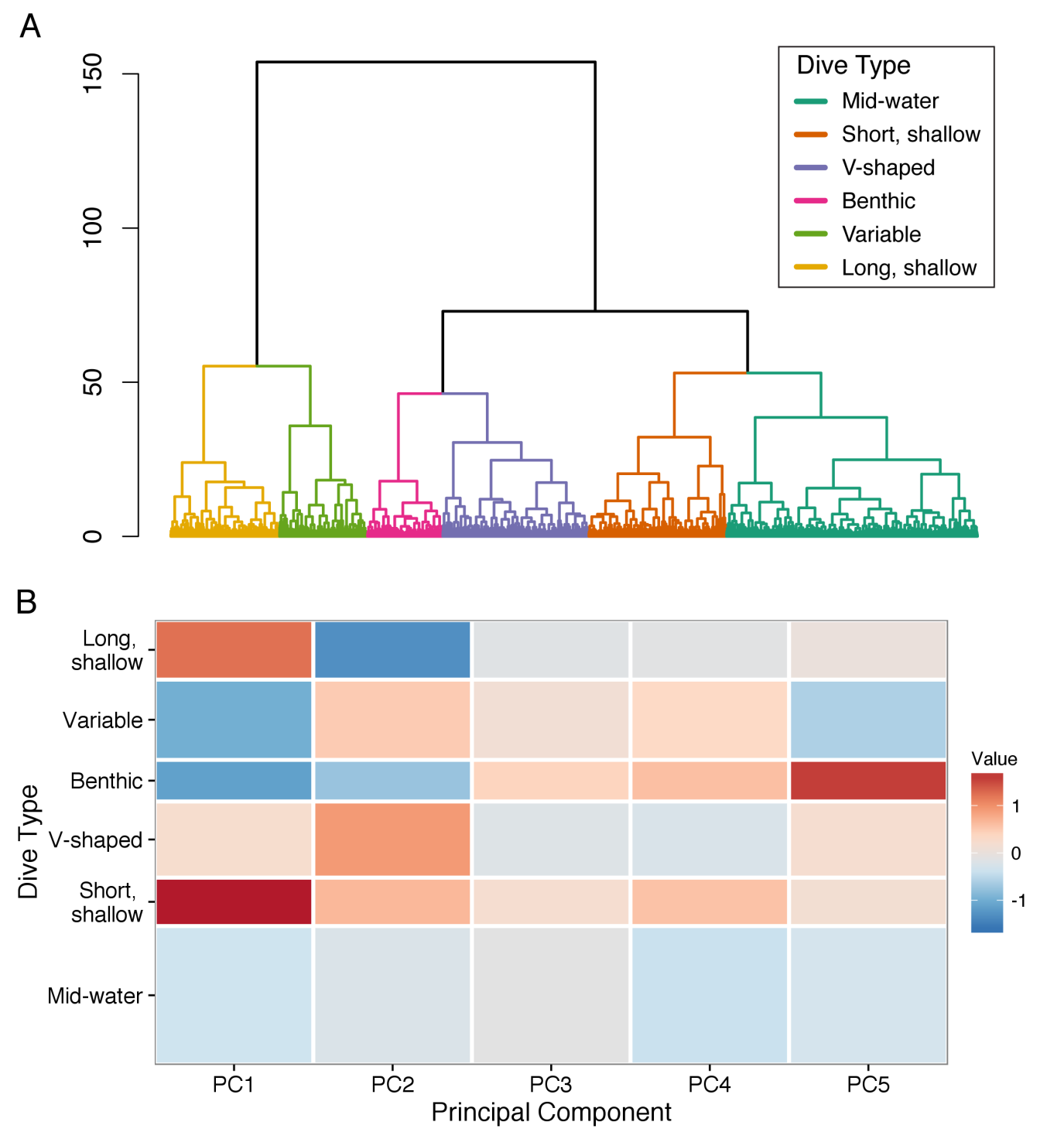


Supplemental Figure S2: A) Dendrogram from a hierarchical cluster analysis using the first five components from a principal components analysis of 12 ADB-tag-derived dive behavior variables as inputs (see Table 1). B) The proportional influence of each principal component (listed in Table 4) on each dive type category identified in the cluster analysis. Colors represent the average value of the rotated data (i.e., the centered and scaled data multiplied by the loadings matrix) for each dive type category from the cluster analysis (tile size is scaled to cluster group size along the y axis; see Table 5). For example, PC1 contrasts long-duration, deep dives and short duration, shallow dives (Table 4). Correspondingly, the two shallow dive types show a positive score (red) while Benthic, Variable, and Mid-water dive types show negative scores (blue).


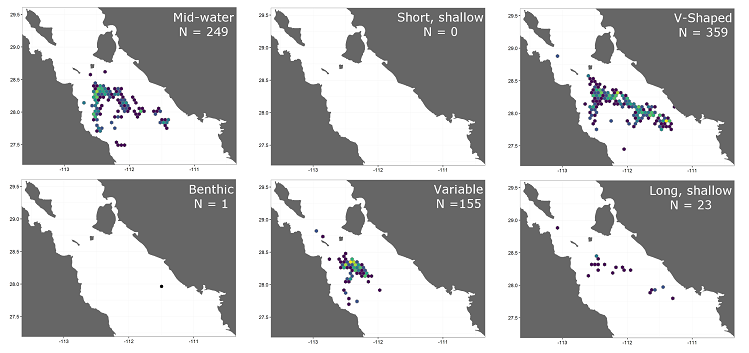


Supplemental Figure S3: Density of occurrence for each of six dive types derived from Argos-transmitted dive summary messages from ADB tags deployed on sperm whales in the Gulf of California during spring 2007 and 2008. A 5-km hexagonal grid was used, with lighter colors representing a higher density of dives in that cell (range 1 – 15). Results were log transformed to better visualize spatial variability of dive density.


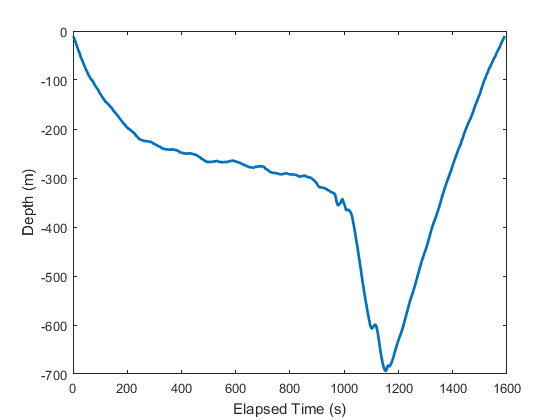


Supplemental Figure S4: Example of a single 27-min Variable type dive that shows the tagged whale diving to a mid-water depth (200-300 m), before rapidly descending to a depth of 700 m over approximately 4 min.
